# Dynamic facial movements shape how confident speakers appear

**DOI:** 10.1098/rspb.2026.2044

**Published:** 2026-10-21

**Authors:** Jonas Nölle, Yichen Wu, Pablo Arias-Sarah, Oliver G. B. Garrod, Philippe G. Schyns, Rachael E. Jack

**Affiliations:** ^1^ School of Psychology & Neuroscience, University of Glasgow, Glasgow, UK; ^2^ Centre for Language Evolution Studies, Nicolaus Copernicus University in Toruń, Torun, Kuyavian-Pomeranian Voivodeship, Poland; ^3^ Department of Cognitive Science, Lund University, Lund, Skåne County, Sweden; ^4^ Psychology and Neurocognition Lab, CNRS Université Grenoble Alpes, Saint-Martin-d'Hères, Auvergne-Rhône-Alpes, France; ^5^ College of Connected Computing, Vanderbilt University, Nashville, TN, USA

**Keywords:** speech comprehension, multimodal communication, social signals, confidence perception, cross-cultural communication, reverse correlation, facial movements

## Abstract

Human communication is inherently multimodal, relying on signals conveyed through speech, vocal tone and facial movements. While gestures and vocal cues are known to influence how speech is understood, the role of facial movements remains poorly specified. Here, we test whether dynamic facial movements shape how spoken responses are interpreted by systematically modulating listener inferences of speaker confidence and doubt. Using a data-driven reverse-correlation approach and a generative model of facial behaviour, we manipulated speakers’ upper-face movements while holding verbal content constant. On each trial, participants (*n* = 44; UK English and Mandarin Chinese speakers) rated the confidence of speakers responding to questions (e.g. ‘Would they make a good leader?’) on a five-point scale across 1800 trials. We find that opposing facial movements bias these inferences: eye closure and brow raising are associated with confidence, whereas brow lowering and eye tightening are associated with doubt. Signal amplitude further shapes these inferences, primarily by intensifying perceived doubt for doubt-associated movements. These mappings are largely shared between the two cultural groups, indicating common signal–inference relationships alongside differences in the weighting of specific cues. Together, these findings show that facial movements provide graded social signals that systematically shape how spoken responses are interpreted.

## 1. Background

Understanding spoken language requires more than decoding words alone. Human communication is inherently multimodal, relying on signals from speech, vocal tone, facial movements and gestures to support mutual understanding [1–8]. When interpreting speech, listeners integrate information not only from what is said but also from how it is expressed [9,10]. For example, when asked, ‘do you know where the train station is?’, a person responding ‘yes’ or ‘no’ firmly may be perceived as more confident than one who responds hesitantly [11–13]. Such expressions of confidence or doubt provide important information that shapes how spoken messages are interpreted, guiding inferences about a speaker's knowledge and reliability [14,15].

Despite the importance of these inferences, most work has focused on how they are conveyed through linguistic and vocal cues. Linguistic markers such as ‘uhm’ and ‘maybe’, as well as phonetic features such as high pitch, rising intonation and slower speech rate, are reliably associated with doubt [11,13,16,17]. In contrast, the face has received comparatively little attention in this context, despite observational evidence that speakers routinely display facial movements, such as a puzzled ‘thinking face’ or ‘facial shrug’, when responding to questions [1,4,18–20]. These observations suggest that facial movements may provide information that shapes how spoken responses are interpreted [21]. This relative neglect reflects, in part, how facial movements have most often been studied—primarily as signals of emotion [22–25] or as behaviours peripheral to speech, rather than as dynamic signals that actively shape the interpretation of speech [26,27]. Crucially, such conversational facial signals need not be dedicated markers of confidence: many of the movements involved (brow raising, brow lowering and eye widening or narrowing) also occur in facial expressions of emotion [28], so their communicative role cannot be read off their morphology but must be established by testing what they do when they accompany speech.

Recent work has begun to examine how specific facial cues contribute to conversational understanding, showing that naturalistic eyebrow movements facilitate question identification [29], and that combinations of conversational facial signals form compositional meanings that shape the interpretation of speaker intentions [30]. Together, this growing literature establishes facial behaviour as an active contributor to conversation, supporting a range of social actions and speaker intentions [29,31–33]. However, these studies examined a small number of pre-specified cues, leaving open which dynamic facial movements shape the interpretation of concurrent speech more generally and how strongly and consistently they do so across individuals. A systematic, data-driven account sampling the space of facial movements rather than testing pre-specified cues is therefore still lacking.

Here, we address this gap by experimentally manipulating the upper-face movements of speakers while they respond to questions and testing whether these signals influence how speech is understood. We focus on perceived speaker confidence and doubt as a tractable instance of this broader function. Throughout, we use ‘understanding’ to refer to listeners’ evaluation of a spoken response—specifically whether the speaker's answer appears confident or doubtful—rather than comprehension of its propositional content.

To do so, we used a generative model of human facial behaviour [34] to agnostically sample facial movements, combined with reverse-correlation methods widely used across ethology, vision science [27,35,36] and neuroscience [37–42], to estimate the relationship between dynamic facial signals and listener judgements. Figure 1 illustrates the approach. We focus on perceived speaker confidence and doubt, that is a speaker's ‘feeling of knowing’ [14,15]. A listener's interpretation of a spoken answer depends on how confident the speaker appears, making it a well-defined and consequential instance of speech interpretation. Interlocutors actively use it to weigh each other's confidence when coordinating and reaching joint decisions [44–46] while keeping both the manipulated signal (upper-face movements) and the measured inference tightly specified.

**Figure 1.**
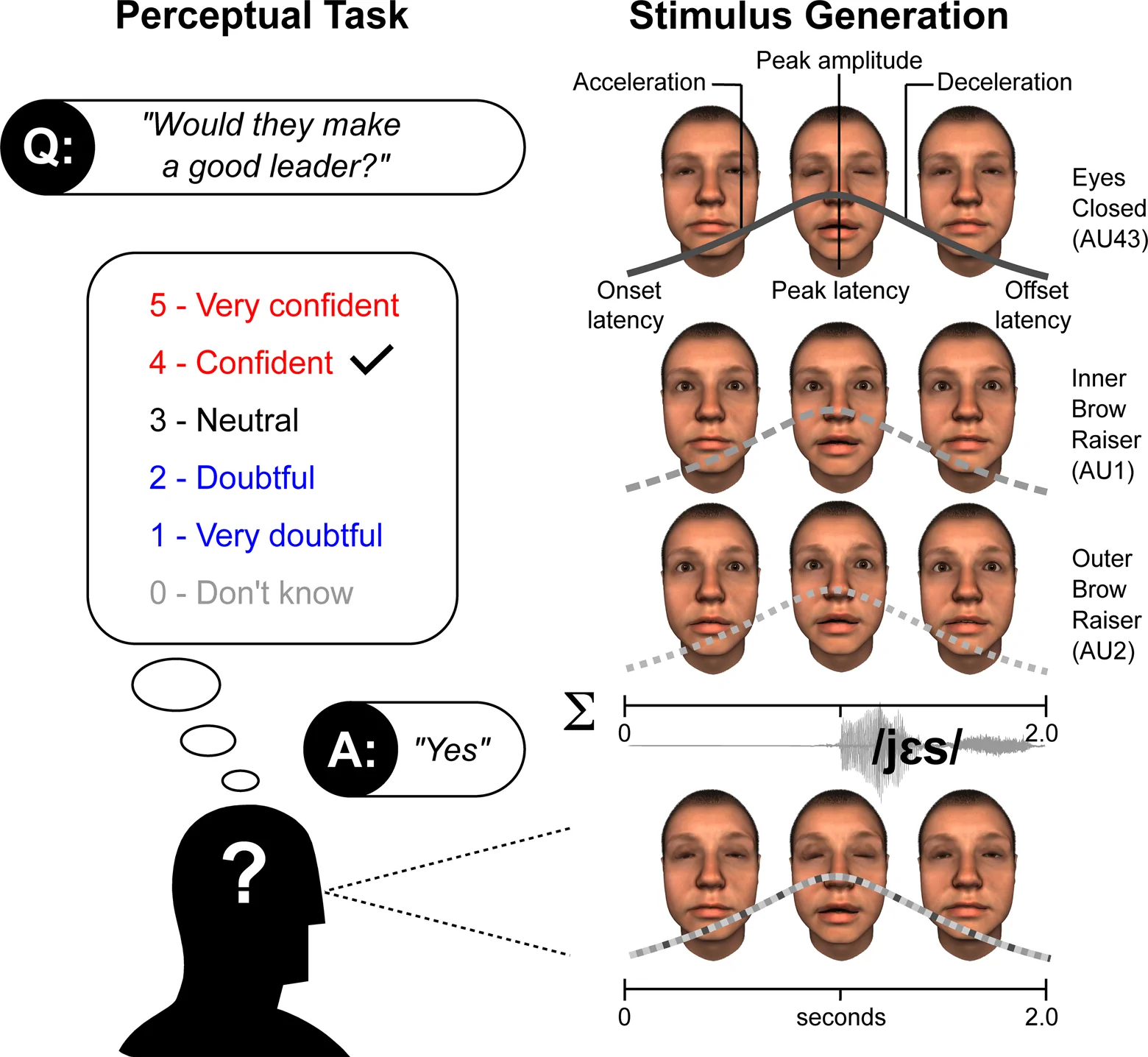
Perceptual task and stimulus generation. In each trial, participants viewed a speaker answering ‘yes’ or ‘no’ to a question (e.g. ‘would they make a good leader?’). While responding, the speaker displayed a facial movement composed of a combination of randomly sampled facial movements, known as action units [AUs; 43]. In the example shown, three AUs are activated: eyes closed (AU43), inner brow raiser (AU1) and outer brow raiser (AU2). All AUs within a trial had the same amplitude, which varied across trials. These movements were synchronized with the onset of the spoken response. After viewing each stimulus, participants rated how confident the speaker's answer appeared on a five-point scale from ‘very doubtful’ to ‘very confident’ or selected ‘don't know’ if unsure. In this example, the participant rated the speaker as ‘confident’ (check mark). Participants in each culture completed 600 trials for each of three question types (1800 trials total), with speakers representing same-ethnicity male and female identities.

Our approach is data-driven [27]: rather than averaging responses across participants, we first estimate a participant-specific model identifying the facial movements associated with each participant's confidence and doubt judgements. This preserves individual variation, enables the consistency of these perceptual mappings to be assessed across participants and provides the basis for Bayesian population-prevalence inference, which estimates how widely the observed mappings generalize across the sampled population rather than averaging individual variation away (see §2). Finally, we test two cultural and linguistic groups – UK English speakers and Mandarin Chinese speakers – because the perception of facial movement signals can vary across cultures [28,47], and because broadening the cultural base of psychological science is itself valuable [48]. We note, however, that both groups are young, university-educated samples from relatively wealthy, industrialized societies, so the commonalities and differences we report need not extend to the full range of human cultures [49]. The two groups nonetheless differ markedly in cultural and linguistic background: the East Asian participants had recently arrived in the United Kingdom and were screened for minimal exposure to non-East-Asian cultures, and the two groups are known to differ in how they perceive facial movements [28,47].

By randomly sampling upper-face movements and varying their amplitude while holding spoken content constant (figure 1), we tested how specific facial signals influence judgements of speaker confidence. This design allows us to estimate how individual facial movements systematically shape interpretation while holding the spoken content constant.

## 2. Methods

### (a) Participants

We recruited 22 White British native English speakers (mean age = 22.3 years, s.d. = 3.3 years; 10 women, 10 men, 2 non-binary/undeclared) and 22 Chinese native Mandarin speakers of East Asian ethnicity (mean age = 24.4 years, s.d. = 3.3 years; 11 women, 11 men). All participants reported normal or corrected-to-normal vision and no psychiatric, neurological or developmental conditions affecting visual perception, face processing, language, hearing or social communication. To minimize prolonged cultural exposure effects, all East Asian participants had resided in the United Kingdom for less than three months and reported minimal exposure to non-East-Asian cultures according to established screening criteria [47]. Participants received £6/hour for participation.

### (b) Facial stimuli

Stimuli comprised 1800 videos of speakers answering ‘yes’ or ‘no’ while displaying facial movements synchronized with speech onset (figure 1; see supplementary material for example videos). We generated speaker identities using a high-fidelity three-dimensional generative model of the human face [34,50]. For each stimulus, we randomly generated a same-ethnicity face identity (18–26 years; sex-balanced).

Each speaker displayed a biologically plausible combination of 1–4 upper-face action units (AUs), randomly sampled from a set of 12 AUs using a binomial distribution (*p* = 0.5, *n* = 4). We restricted the manipulation to the upper face because many conversational facial signals (e.g. the ‘thinking face’ and ‘facial shrug’) also recruit lower-face movements, which would interfere with mouth movements and the acoustic properties of speech. On each trial, all AUs had the same amplitude, which varied across five levels (0.2, 0.4, 0.6, 0.8 and 1.0) across trials. Additional animation and AU details are provided in the supplementary material.

### (c) Voice and lip movements

To ensure that facial movement effects were independent of vocal identity, we paired facial stimuli with 20 computer-transformed voice identities derived from recordings of native English speakers and native Mandarin speakers. We parametrically manipulated vocal pitch and spectral envelope while preserving linguistic content and intonation, generating naturalistic voice identities that differed in speaker characteristics but not semantic content. We synchronized lip movements with speech using VOCA-generated facial animation [51]. Additional voice generation and transformation details are provided in the supplementary material.

### (d) Multimodal stimuli

To generate the multimodal speaker stimuli, we randomly paired 1 of the 20 voice identities with 1 of the 1800 unique face identities, evenly distributed across trials. Each voice identity therefore appeared 90 times during the experiment. To synchronize the visual and auditory channels, we aligned the onset of each spoken utterance (e.g. ‘yes’ or ‘no’) with the peak amplitude of the facial movements (1 s after stimulus onset; figure 1). Each stimulus had a total duration of 2 s.

### (e) Question type

We included three question types adapted from previous work [13]: ‘Would they make a good leader?’, ‘Did they complete their application?’ and ‘Will they finish the essay tonight?’. These questions corresponded to distinct communicative functions – making a judgement, stating a fact and expressing an intention, respectively – and allowed us to test the generality of facial movement effects across conversational contexts [52].

For Mandarin-speaking participants, we adapted the questions to enable balanced bi-syllabic responses using 是的 (shì de) (‘yes’, ‘that's right’ or ‘correct’) and 不是 (bú shì) (‘no’, ‘is not’ or ‘that's not the case’). Specifically, the questions were presented as 他是不是一个好的领导者? (Tā shì bú shì yí ge hǎo de lǐngdǎozhě?) (‘Is he a good leader?’), 他是不是在申请工作? (Tā shì bú shì zài shēnqǐng gōngzuò?) (‘Is he applying for jobs?’) and 他是不是想今晚完成他的论文? (Tā shì bú shì xiǎng jīnwǎn wánchéng tā de lùnwén?)(‘Does he want to finish his dissertation tonight?’). In spoken Mandarin, the pronoun *tā* is phonetically identical for ‘he’ and ‘she’, and the written character 他 is commonly used as a gender-neutral third-person form in conversational contexts.

### (f) Task procedure

Following four practice trials, participants completed 1800 experimental trials across three sessions. On each trial, participants viewed a speaker responding ‘yes’ or ‘no’ while displaying a facial movement synchronized with the utterance (figure 1). Participants then rated how confident the speaker's response appeared using a five-point scale ranging from ‘very doubtful’ to ‘very confident’. Participants selected ‘neutral’ if the response appeared neither confident nor doubtful and ‘don't know’ if none of the response options adequately captured their perception. We instructed participants to respond quickly and avoid overthinking their judgements. Trials were grouped by question type and stimulus-face sex, and block order was randomized across participants.

### (g) Modelling facial movements representing speaker confidence and doubt

To examine whether facial movements influenced how participants interpreted speaker confidence, we estimated, for each participant, the statistical relationship between the randomly sampled AUs presented on each trial and the participant's responses on the bipolar confidence–doubt scale. We excluded all ‘don't know’ responses from analysis (Western European participants: mean = 3.1% of trials, range = 0–11%; East Asian participants: mean = 3.3%, range = 0–12%) and pooled the remaining trials across the three question types for each participant (see supplementary material: testing influence of question type, stimulus sex and answer type; figure S4).

For each participant, we fitted ordinal cumulative link models (CLMs) with a logit link function to estimate the relationship between individual AUs and the latent continuous response variable [53]. Models additionally included question type, answer type (‘yes’ or ‘no’) and the interaction between utterance pitch and stimulus-face sex.

We established statistical significance using a non-parametric permutation procedure. For each participant, we randomly shuffled response labels and refitted the same CLM across 1000 iterations, generating null distributions of beta coefficients for each AU. We classified AUs as significantly associated with perceived confidence or doubt when empirical beta coefficients fell outside of the 2.5th–97.5th percentiles of the permuted distributions (two-tailed *p* < 0.05). These individual-level permutation tests were evaluated separately for each AU and were not corrected for the number of AUs tested within a participant. Rather than correcting for this multiplicity at the individual level, our inference relies on Bayesian population-prevalence analysis, which estimates how widely effects replicate across participants. The prevalence threshold (at least 4 of 22 participants) therefore guards against isolated false positives arising through multiple testing within individual participants.

Applying this procedure to each participant yielded 22 facial movement models of confidence and 22 models of doubt per culture (88 models in total). Whereas facial movement effects remained robust across analyses, effects of question type, answer type, stimulus-face sex and voice pitch did not exceed statistical thresholds (see also supplementary material, figures S4–S6).

### (h) Validating participant-specific facial movement models of speaker confidence and doubt

Before drawing population-level inferences, we evaluated whether the participant-specific facial movement models captured reliable and discriminable mappings between facial movements and confidence versus doubt judgements. This validation step establishes the robustness of the participant-specific models. We validated the resulting facial movement models using a machine learning approach. For each culture separately, we trained naive Bayes classifiers to distinguish confidence from doubt using the facial movement models derived above, represented as binary vectors of statistically significant AUs. We evaluated all 88 models using leave-one-out cross-validation. We retained for subsequent analyses only the participant-specific models that both exceeded the classification threshold and were classified correctly. Applying these criteria retained 81 of the 88 models: for Western European participants, all 22 confidence models and 21 of 22 doubt models; for East Asian participants, 21 of 22 confidence models and 17 of 22 doubt models.

Additionally, as a stronger test, we assessed whether each participant's facial-signal-to-judgement mapping predicted their responses on held-out trials. For each participant, we entered all 12 AUs as predictors, without feature selection, and used five-fold cross-validation to predict confidence versus doubt on trials not used for fitting. Significance was established with the same permutation procedure used above, shuffling response labels within participants and re-running the full cross-validation across 1000 iterations. Held-out prediction was significantly above chance for 22 of 22 Western European participants (mean AUC = 0.83) and 21 of 22 East Asian participants (mean AUC = 0.79), in both cultures well exceeding the Bayesian population-prevalence threshold (at least 4 of 22). The recovered mappings therefore generalize to new trials rather than simply redescribing the data from which they were estimated.

### (i) Bayesian population prevalence

To obtain population-level inference from the within-participant analyses, we used Bayesian population prevalence [54]. This approach estimates the proportion of the population expected to show a true positive effect for a given test (e.g. an association between a specific AU and perceived confidence) and tests the null hypothesis that the population prevalence equals zero.

Given 22 participants per culture and a significance threshold of *p* < 0.05, the null hypothesis could be rejected when significant within-participant effects were observed in at least 4 of the 22 participants. We therefore retained for further analyses only AUs exceeding this prevalence threshold (4/22 participants). Figure S1 in the supplementary material shows the corresponding unthresholded results.

### (j) Measuring facial movement specificity for confidence and doubt

To determine whether specific AUs were selectively associated with confidence or doubt, we quantified the relationship between each AU and response category using mutual information (MI) [55,56]. Specifically, we calculated the MI between each AU and the confidence–doubt classification separately for each culture. Higher MI values indicate stronger selectivity for one response category, whereas lower values indicate that an AU contributed similarly to both confidence and doubt.

To establish statistical significance, we used a non-parametric permutation procedure. For each culture, we randomly shuffled the confidence and doubt labels across AUs and recomputed MI values across 1000 iterations, generating a null distribution for each AU. We compared observed MI values against the 95th percentile of the corresponding null distributions (one-tailed *p* < 0.05). AUs exceeding this threshold were classified as selectively associated with confidence or doubt; otherwise, they were considered shared across both response categories. We applied this procedure separately to each participant in each culture. Figure S2 in the supplementary material shows the resulting specificity patterns.

### (k) Measuring cultural specificity of facial movements

To determine whether facial movements associated with confidence and doubt were culture-specific or shared across cultures, we applied the same MI analysis described above. Specifically, we calculated the MI between each AU and participant culture (Western European versus East Asian) separately for confidence and doubt responses (i.e. MI [AU, culture]). Statistical significance was established using the same non-parametric permutation procedure, in which culture labels were randomly shuffled across AUs. Figure S3 in the supplementary material shows the resulting cultural-specificity patterns.

### (l) Analysis of facial movement amplitude

To examine how the amplitude of facial movements shaped confidence and doubt judgements in each culture, we analysed trials containing confidence-associated and doubt-associated AUs separately. Each trial contained one to four AUs. A trial was classified as confidence-associated if it contained at least one of the participant's validated confidence-associated AUs and none of their validated doubt-associated AUs; doubt-associated trials were classified analogously. Among trials with ‘confident’ or ‘doubtful’ responses, we excluded trials containing both confidence- and doubt-associated AUs (Western European: 9784 of 33 765 trials, 29.0%; East Asian: 11 555 of 31 161 trials, 37.1%), as well as trials containing no validated confidence- or doubt-associated AUs. For each trial type, we modelled the proportion of ‘confident’ versus ‘doubtful’ responses across the five AU-amplitude levels. We fitted linear models to these participant-level proportions, weighting each participant equally. As response proportions are bounded and the number of trials per amplitude level varied across participants, we confirmed these effects using mixed-effects logistic regression on trial-level responses, with amplitude as a fixed effect and by-participant random intercepts and slopes for amplitude.

### (m) Software

We conducted all analyses using R v.4.0.5 [57] and MATLAB 2020b (see supplementary material for a full list of software libraries).

## 3. Results

We modelled and validated the dynamic facial movements associated with perceived speaker confidence and doubt for each participant. Facial movements systematically influenced how participants interpreted speakers’ responses. Across cultures, specific movements shifted judgements of confidence and doubt in consistent and opposing directions. Figure 2 shows these effects aggregated across participants, including all facial movements that exceeded the population-prevalence threshold (*n* = 4; see supplementary material, figure S1 for unthresholded results).

**Figure 2.**
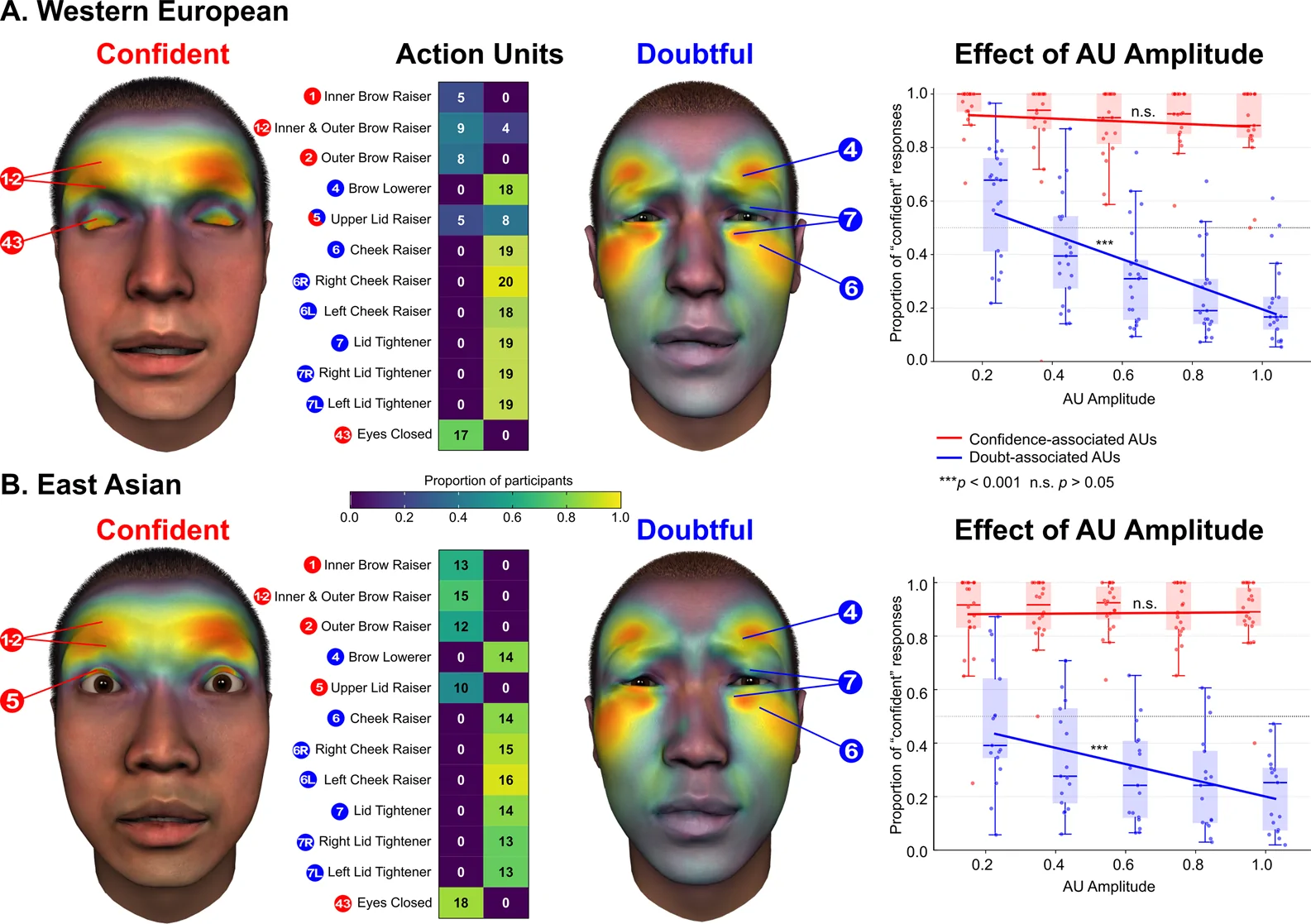
Facial movements associated with the perception of speaker confidence and doubt. Results are shown separately for Western European (A) and East Asian (B) participants. Face maps (left) display the facial movements (action units, AUs) significantly associated with perceived confidence (left column) and doubt (right column). Colour-coding reflects the magnitude of deviation from a neutral face at peak amplitude, and facial locations are labelled by AU number. The central matrix shows the proportion of participants exhibiting each effect, with warmer colours indicating higher prevalence. Across cultures, confidence is primarily associated with brow raising (AU1–2) and eye closure (AU43), whereas doubt is associated with brow lowering (AU4) and eye tightening (AU6–7). Cultural differences are evident in the use of eye widening (AU5), which is associated with both confidence and doubt in Western Europeans but more strongly with confidence in East Asians. Box plots (right) show the proportion of responses classified as ‘confident’ as a function of AU amplitude, plotted separately for trials containing only confidence-associated (red) or only doubt-associated (blue) movements; points show individual participants and lines show the fitted trend.

### (a) Specific facial movements shape perceptions of speaker confidence

In both Western European and East Asian participants, confidence was primarily associated with eyebrow raising (AU1–2; Western European: *n* = 5–9 of 22 participants; East Asian: 12–15 of 21 participants) and eye closing (AU43; Western European: *n* = 17 of 22 participants; East Asian: *n* = 18 of 21 participants), whereas doubt was associated with brow lowering (AU4; Western European: *n* = 18 of 21 participants; East Asian: *n* = 14 of 17 participants) and squinting (AU7; Western European: *n* = 19 of 21 participants; East Asian: *n* = 14 of 17 participants; AU6; Western European: *n* = 19 of 21 participants; East Asian: *n* = 14 of 17 participants) (figure 2). Mutual information analysis confirmed that these facial movements showed selective associations with confidence and doubt in each culture (supplementary material, figure S2). Cross-cultural analyses further revealed broadly shared mappings across cultures, with one notable difference: East Asian participants associated eye widening (AU5) more strongly with confidence (*n* = 10/21), whereas Western European participants associated AU5 with both confidence and doubt (*n* = 5/22, *n* = 8/21, respectively; supplementary material, figure S3). Thus, these findings show that distinct facial movements systematically shape perceptions of speaker confidence and doubt across cultures.

### (b) Facial movement amplitude selectively modulates judgements of speaker doubt

Within doubt-associated AUs, the proportion of confident responses fell steeply with increasing AU amplitude across participants (Western European: 0.61–0.22; East Asian: 0.47–0.21; both *p* < 0.001), whereas within confidence-associated AUs, it remained high across the amplitude range (Western European: 0.95–0.88, *p* = 0.33; East Asian: 0.87–0.88, *p* = 0.85; see figure 2, right). Trial-level mixed-effects logistic regressions confirmed this pattern: increasing amplitude reduced confidence responses for doubt-associated AUs (Western European: *β* = −2.449, *p* < 0.001; East Asian: *β* = −1.877, *p* < 0.001), but not for confidence-associated AUs (Western European: *β* = −0.495, *p* = 0.303; East Asian: *β* = −0.343, *p* = 0.359). Thus, amplitude mattered specifically for judgements of speaker doubt: the stronger a doubt-associated facial movement, the more doubtful the speaker appeared, whereas confidence-associated movements continued to elicit predominantly confident judgements across the tested amplitude range.

Together, these results show that specific and opposing facial movements systematically shape how listeners interpret spoken responses across cultures, with largely shared, graded effects on perceived confidence and doubt.

## 4. Discussion

Here, we show that facial movements systematically shape how listeners interpret spoken responses. By experimentally manipulating facial signals while holding verbal content constant, we provide causal evidence that specific movements modulate interpretation in structured and opposing ways. Across cultures, brow raising and eye closure were associated with increased perceived confidence, whereas brow lowering and eye tightening signalled doubt. These findings extend previous observational accounts of facial signals in conversation by demonstrating that such movements do not merely accompany speech but actively shape how it is understood. In particular, the strong effect of eye closure is consistent with prior work suggesting that prolonged eye-blinks can signal understanding in interaction [58], while the role of brow lowering aligns with descriptions of the ‘facial shrug’ as a marker of uncertainty [18]. Crucially, our findings show that these signals operate within a multimodal system, in which facial movements alter how spoken responses are interpreted even in the absence of prosodic cues to confidence.

Our findings also place these confidence and doubt signals within the broader repertoire of human facial expression. The recovered configurations overlap in part with canonical emotion expressions: brow raising and eye widening as in surprise, brow lowering with lid tightening as in anger or concentration. Under a view of the face as a dynamic tool for social communication [26,27], such overlap is expected. A common set of AUs is reconfigured to transmit different social messages, recurring across emotional, conversational and even grammatical signals [28,59], and several are grounded in biologically rooted movements that regulate sensory intake and signal approach or rejection [60,61]. In this view, the meaning of a movement is not fixed by its morphology but resolved by its context, here a concurrent spoken answer. The question is therefore not whether there is a dedicated ‘confidence face’, but how movements drawn from this shared repertoire come to convey confidence and doubt when they accompany speech. What our data add is evidence that these movements causally shape how a concurrent spoken answer is interpreted. Whether receivers reach this inference by first recognizing a familiar affective state or read confidence more directly remains an open question – and the same data-driven approach can address it, for example by modelling emotion and confidence judgements of the same stimuli.

We also observed systematic cultural differences in how these facial signals were weighted. Although brow raising was consistently associated with confidence in both groups, its influence on interpretation varied across cultures. Western European participants more strongly associated squinting movements with doubt, whereas East Asian participants relied more heavily on brow raising as a marker of confidence. A further difference emerged for eye widening (AU5), which East Asian participants associated more consistently with confidence, while its interpretation was less stable among Western European participants. These findings suggest that while the underlying mapping between facial movements and interpretation is broadly shared, cultures calibrate the relative importance of specific cues. Such differences may reflect variation in the pragmatic roles of facial signals in communication, including their use for emphasis or social signalling [19,62]. The clearest divergence, East Asian observers’ more consistent reading of eye widening as confident, is compatible with eye widening being a highly salient cue that cultures weight differently. As this comparison spans only two groups, however, such differences are best interpreted as culturally modulated tendencies rather than fixed cultural signatures.

Finally, the effect of facial movement amplitude depended on the signalling role of the movement rather than simply the overall amount of facial movement. Increasing amplitude intensified the doubt conveyed by doubt-associated movements, whereas confidence-associated movements continued to elicit high confidence judgements across the tested amplitude range. These findings reveal an asymmetry in the observed amplitude effects: increasing amplitude graded judgements of doubt, whereas confidence-associated movements elicited high confidence judgements across the tested amplitude range. This pattern could reflect a difference in how the two inferences are encoded, with doubt signalled parametrically and confidence more categorically, though the high confidence judgements already observed at low amplitude also leave open a ceiling-effect explanation. Graded signalling of doubt is functionally useful because it gives receivers a continuous cue to how far to trust a speaker's answer and how heavily to weight it when coordinating a decision [44–46]. Distinguishing between these possibilities will require testing confidence-associated movements over a range that avoids ceiling performance. This pattern also argues against a simple naturalness account, under which high-amplitude faces would be discounted as exaggerated – such an account would predict reduced confidence for high-amplitude confidence-associated movements, yet confidence-associated movements continued to convey confidence across the tested amplitude range. The graded effect of amplitude on doubt is consistent with a parametric signalling system in which receivers jointly decode a movement's identity and its intensity [41]. Although restrained vocal delivery is often perceived as confident [13], our results suggest this reflects the graded strength of specific doubt-related cues rather than a general link between expressive restraint and confidence. Together, these findings suggest that listeners integrate facial information continuously when interpreting spoken responses, rather than relying on isolated or stereotyped expressions.

Our approach also bounds these conclusions and motivates further work. We estimated each movement's contribution additively while holding the co-occurring movements constant. Given that the diagnostic value of an AU may depend on the ensemble of movements in which it appears, this approach recovers movements with a consistent directional effect but may underestimate cues whose diagnostic contribution is context dependent. The movements identified here are therefore best read as a lower bound on the facial information that shapes these inferences, and mapping their configural interactions is a natural extension of the data-driven approach used here.

These findings open several avenues for future work. A natural next step is to manipulate facial and vocal cues together in a combined reverse-correlation design, to quantify how visual and acoustic signals are weighted and integrated when they match or conflict. Extending the approach to a wider range of conversational inferences and to more diverse cultural and linguistic populations would further test the generality of these signal-to-inference mappings. More practically, a precise, causal account of how facial movements convey confidence can inform the design of socially interactive virtual agents that express and elicit confidence appropriately during human interaction.

Taken together, our findings demonstrate that facial movements systematically shape how listeners interpret spoken responses. By showing that specific facial signals exert structured and opposing effects on these judgements across cultures, with signal amplitude further grading perceived doubt, this work extends current accounts of multimodal communication beyond linguistic and vocal cues alone. More broadly, these findings highlight the need for theories of language and social communication to account for the dynamic contribution of facial behaviour to speech interpretation.

## Supplementary Material



## Data Availability

Behavioural data, analysis code and example stimuli are available at the Open Science Framework repository [63]. Supplementary material is available online [64].
