# Supplementary material for: Dynamic facial movements shape how confident speakers appear
Source: Proc Biol Sci. 2026 Oct 21;293(2081):20262044. doi: 10.1098/rspb.2026.2044 (PMC13682920; doi:10.1098/rspb.2026.2044)
Supplement: Supplementary file 1 [file rspb.2026.2044.zip › rspb.2026.2044/rspb20262044_si_001.pdf]

Electronic Supplementary Material

Dynamic facial movements shape how confident speakers appear

Jonas Nölle, Yichen Wu, Pablo Arias-Sarah, Oliver G. B. Garrod, Philippe G. Schyns, and Rachael E. Jack

Proceedings of the Royal Society B: Biological Sciences

Article DOI: 10.1098/rspb.2026.2044

Stimuli

Face identities and facial movements

For each trial, we generated a random same-ethnicity face identity (male or female; 18–26 years) using a high-fidelity 3D generative model derived from captured human faces (1, 2). Each face displayed a random biologically plausible combination of 1–4 upper-face Action Units (AUs), sampled from 12 possible AUs using a binomial distribution ($n = 4$, $p = .5$). We restricted AUs to the upper face to avoid interference with speech-related mouth movements.

The AU set comprised AU1 (Inner Brow Raiser), AU2 (Outer Brow Raiser), AU1+2 (Inner Brow Raiser + Outer Brow Raiser), AU4 (Brow Lowerer), AU5 (Upper Lid Raiser), AU6 (Cheek Raiser), AU6L (Cheek Raiser Left), AU6R (Cheek Raiser Right), AU7 (Lid Tightener), AU7L (Lid Tightener Left), AU7R (Lid Tightener Right), AU43 (Eyes Closed).

We animated all AUs using identical temporal parameters (onset latency, acceleration, peak amplitude, peak latency, deceleration, offset latency; see Fig. 1) implemented using cubic Hermite spline interpolation (1). AU amplitude varied randomly across five levels (0.2, 0.4, 0.6, 0.8, 1.0).

Voices and lip movements

To generate vocal identities, we recorded two native English speakers (1 female, 1 male) producing "yes" and "no," and two native Mandarin speakers (1 female, 1 male) producing 是的 ("yes") and 不是 ("no"). Recordings were obtained using Audacity, a Focusrite Scarlett 2i2 digital interface, and a Gefell UMT 800 microphone. We normalised recording durations using IRCAM's super-vp algorithm (3, 4).

We transformed each voice by independently shifting pitch (−250 to +250 cents, 50-cent increments) and spectral envelope (−250 to +250 cents, 50-cent increments), generating 20 natural-sounding identities per language (10 females, 10 males). Loudness was normalised using the pyloudnorm Python module (5). From the transformed utterances, we selected the 10 male and 10 female voices judged by the first three authors to sound most naturalistic and qualitatively distinct.

We generated synchronised lip movements from the source voices using VOCA (6). Morphology, pitch contour, duration, and semantic content were held constant across all speech tokens, isolating variation in facial movements during confidence judgments.

Question type manipulation

Mandarin question prompts were adapted to permit bisyllabic responses using **是的** ("yes") and

不是 (“no”). For example, the judgment question “Would they make a good leader?” was presented as 他们会是一个好领导吗？

Task Procedure

Participants completed the experiment in a darkened, sound-controlled room using Sennheiser headphones and a 19-inch Dell monitor (60 Hz refresh rate, 1920 × 1280 resolution). We presented face stimuli centrally on a black background. White faces measured on average 17.83 × 10.79 cm and East Asian faces measured 18.63 × 11.80 cm, subtending visual angles of 14.32° × 8.69° for Western European participants and 14.35° × 9.12° for East Asian participants. Based on established estimates of typical social interaction distance (7) and average human face size (8), these values approximated the visual angle of human faces during everyday social interaction. Viewing distance was held constant using a chin rest (Western European participants: 71 cm; East Asian participants: 74 cm).

Trials were grouped by question type and speaker sex (9), and block order was pseudo-randomised across participants. Each block began with a question displayed for at least 2 s, followed by participant initiation of the block. Each trial consisted of a fixation cross (0.5 s), a 2 s dynamic face stimulus, and a confidence rating screen.

Participants rated how confident the speaker’s response appeared using a 5-point scale or selected a “don’t know” option. The experiment comprised three 1-hour sessions (12 blocks of 50 trials each), with short mandatory breaks every 150 trials and at least 1-hour breaks between sessions.

Statistical Modeling

We excluded “don’t know” responses from analysis (Western European: $M = 3.1\%$; East Asian: $M = 3.3\%$). For each participant, we pooled trials across question types and fitted ordinal cumulative link models (CLMs) with a logit link (10), predicting confidence ratings from the AU set present on each trial and corresponding AU amplitudes.

Models including AU amplitudes outperformed binary AU-presence models (Western European $\Delta AIC = -187.9$; East Asian $\Delta AIC = -103.7$), indicating that both AU identity and movement intensity contributed to confidence judgments.

We assessed statistical significance using 1000-iteration within-participant permutation tests. For each iteration, we shuffled confidence ratings and refitted the CLM to obtain permuted beta distributions. We classified an AU as significant when its empirical beta coefficient fell outside the 2.5^{th}–97.5^{th} percentiles of the corresponding permuted distribution (two-tailed, $p < 0.05$).

Models additionally included question type, answer type, utterance pitch, and stimulus-face sex as covariates. None produced significant effects after permutation testing.

Effect size reporting

We calculated effect sizes as odds ratios (ORs) derived from cumulative link model coefficients ($OR = e^\beta$). OR values greater than 1 indicated that increasing Action Unit intensity increased the likelihood of higher confidence ratings, whereas OR values less than 1 indicated a shift toward doubt. Complete effect-size estimates for all Action Units and both cultural groups are provided as CSV files in the OSF repository.

Validation of Action Unit Profiles

To assess whether participant-level Action Unit (AU) effect profiles reflected coherent and interpretable mappings, we performed a validation analysis at the level of individual models. We trained Naïve Bayes classifiers to categorise the 88 participant-level facial movement models (binary vectors marking significant AUs) as "confidence" or "doubt." Using leave-one-out cross-validation and a conservative posterior threshold of .95, we identified profiles that could be reliably assigned to one of the two response categories. This procedure retained 81 participant-level models for further analysis (Western European participants: 22 confidence, 21 doubt; East Asian participants: 21 confidence, 17 doubt).

Fig. S1 visualises the individual-level AU effect profiles retained following model validation, prior to population-level prevalence estimation and thresholding.

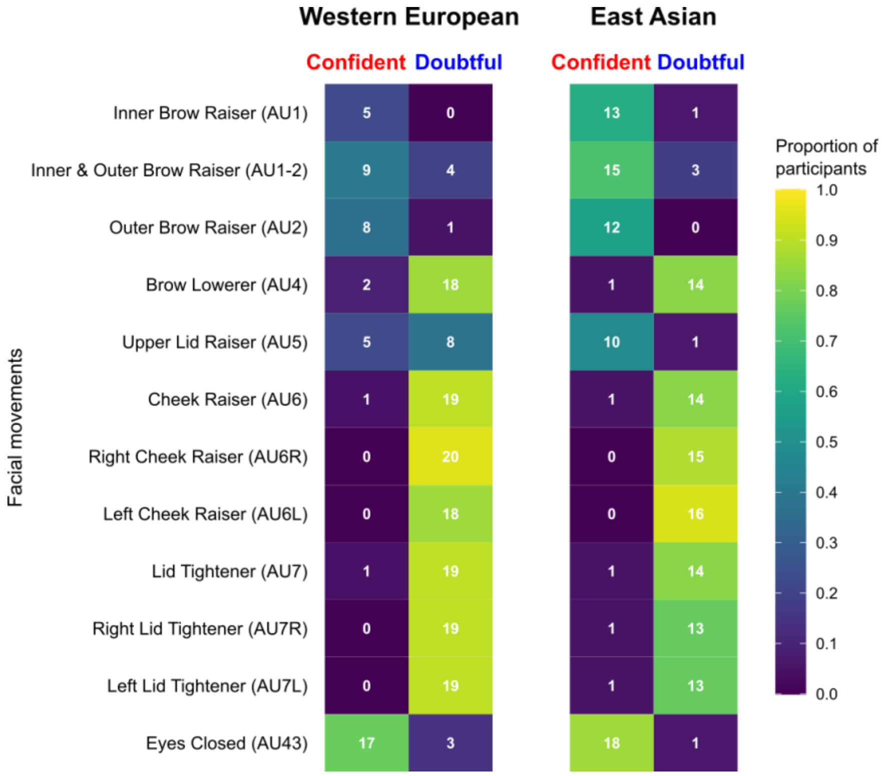

Supplementary Figure S1. Unthresholded individual-level Action Unit (AU) effect profiles prior to population-level inference. For each culture—Western European (left) and East Asian (right)—colour-coded matrices summarise individual-level AU effects on speaker confidence judgements. Each cell indicates the number (and corresponding proportion) of participants whose within-participant model showed a statistically significant association between a given AU and confidence judgements ($p < .05$, permutation test). Warmer colours indicate more participants and cooler colours indicate fewer participants (exact counts shown within each cell). The figure shows the individual-level AU effect profiles retained after model validation, prior to Bayesian population prevalence estimation and thresholding.

Bayesian Population Prevalence

We estimated population-level effects using Bayesian prevalence analysis (11), estimating the population prevalence of participants showing genuine within-participant effects while

accounting for false positives. Under this criterion, effects observed in more than four participants per group were classified as reliable.

Cross-validated prediction
To test whether the participant-specific mappings generalised beyond the trials from which they were estimated, we assessed out-of-sample prediction. For each participant we fitted a logistic model predicting confident (ratings 4–5) versus doubtful (ratings 1–2) responses from all twelve Action Units. All AUs were entered as predictors, with no feature selection, so that held-out predictions were not informed by the significance testing used to define the participant-specific models. Performance was evaluated by five-fold cross-validation, with the area under the receiver operating characteristic curve (AUC) computed on the pooled held-out predictions. Significance was established per participant using a non-parametric permutation test: we shuffled the confidence and doubt labels within participant, re-ran the entire cross-validation procedure across 1000 iterations, and compared the observed AUC against the resulting null distribution (one-tailed $p < 0.05$). Null distributions were centred on 0.50, confirming the procedure was unbiased. Held-out prediction was significantly above chance in 22 of 22 Western European participants (mean AUC = 0.83, range 0.65–0.95) and 21 of 22 East Asian participants (mean AUC = 0.79, range 0.51–0.95); the single non-significant participant had an AUC of 0.51.

Action Unit Specificity (Mutual Information)
We assessed two forms of Action Unit specificity using mutual information (MI; 12, 13):

1. Perceptual Specificity. For each participant, we computed the mutual information between each AU and confidence versus doubt classifications. We generated null distributions by permuting response category labels across 1000 iterations. We classified an AU as perceptually specific when its observed MI exceeded the 95th percentile of the corresponding permuted distribution (one-tailed $p < .05$).

2. Cultural Specificity. We computed the mutual information between each AU and participant culture (East Asian versus Western European) and applied the same permutation-based thresholding procedure.

Figs S2 and S3 visualise perceptual and cultural specificity patterns derived from the mutual information analyses.

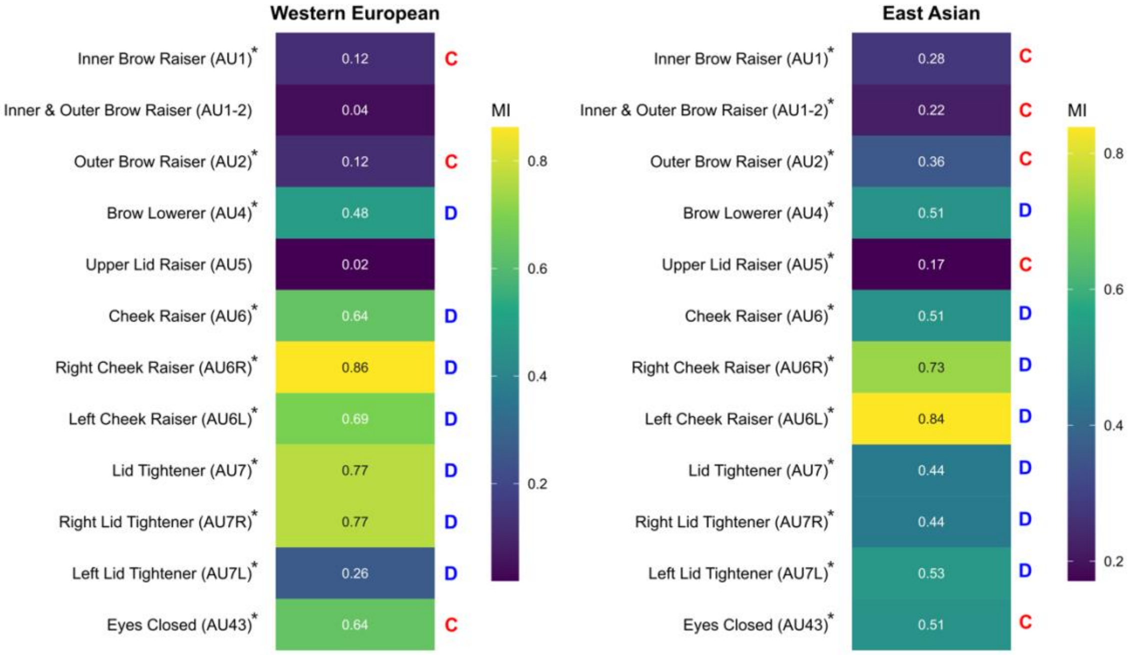

Supplementary Figure S2. Perceptual specificity of facial Action Units.
Colour-coded matrices summarise the perceptual specificity of each facial Action Unit (AU) for distinguishing confidence from doubt within each culture. Each cell reports the mutual information (MI) between AU presence and confidence judgements, with warmer colours indicating higher MI values (greater specificity) and cooler colours indicating lower MI values (exact MI values shown within each cell). Statistical significance was assessed at the individual-participant level using permutation-based null distributions (1000 iterations, $\alpha = .05$). Asterisks indicate AUs whose MI values exceeded the permutation threshold (red “C” = confidence; blue “D” = doubt). Most AUs showed greater specificity for either confidence or doubt, whereas AU1/2 and AU5 showed lower specificity among Western European participants.

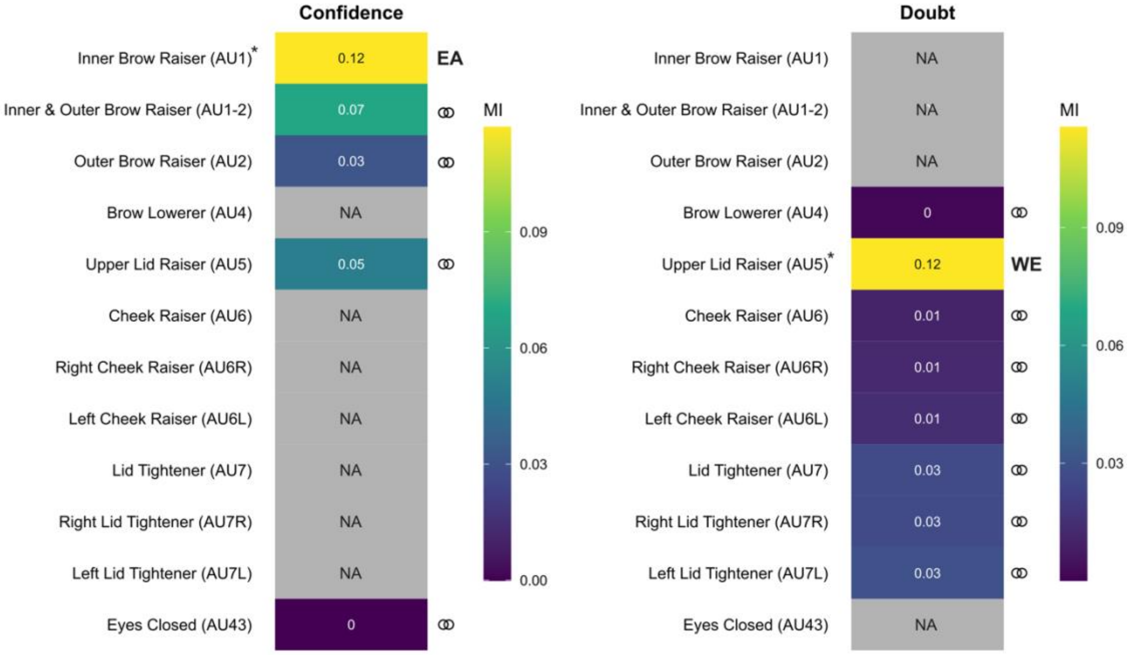

Supplementary Figure S3. Cultural specificity of facial Action Units.
Colour-coded matrices summarise the cultural specificity of each facial Action Unit (AU) separately for confidence and doubt. Each cell reports the mutual information (MI) between AU presence and observer culture (Western European versus East Asian), with warmer colours indicating higher MI values (greater cultural specificity) and cooler colours indicating lower MI values (exact MI values shown within each cell). Statistical significance was assessed at the individual-participant level using permutation-based null distributions (1000 iterations, $\alpha = .05$). Asterisks indicate AUs whose MI values exceeded the permutation threshold ("WE" = Western European-specific; "EA" = East Asian-specific; "⚭" = effects observed in both cultures). Gray "NA" cells indicate AUs that did not exceed the permutation threshold. For confidence judgements, AU1 showed cultural specificity, whereas AU1–2, AU2, AU5, and AU43 showed significant effects in both cultures. For doubt judgements, AU5 showed cultural specificity, whereas AU4, AU6, AU6R/L, and AU7R/L showed significant effects in both cultures.

Facial movement amplitude

To examine how Action Unit (AU) amplitude shaped confidence and doubt judgements, we analysed trials containing confidence-associated and doubt-associated Action Units separately. Each trial contained one to four AUs. A trial was classified as confidence-associated if it contained at least one of the participant's validated confidence-associated AUs and none of their validated doubt-associated AUs; doubt-associated trials were defined analogously. Trials containing both confidence- and doubt-associated AUs, or no validated confidence- or doubt-associated AUs, were excluded from this analysis. For each trial type, we calculated the proportion of responses classified as "Confident" (ratings 4–5) across the five AU-amplitude levels. The resulting response proportions are shown in Figure 2 of the main text. We fitted linear models to these participant-level proportions, weighting each participant equally. Because proportions are bounded and the number of trials per amplitude level varied across participants, we confirmed these effects using mixed-effects logistic regression on trial-level responses, with amplitude as a fixed effect and by-participant random intercepts and slopes for amplitude; this reproduced the same pattern in both cultures.

Software

We conducted all analyses using R 4.0.5 and MATLAB 2020b. In R, we used the following packages: ordinal, tidyverse, MuMIn, sjstats, ggplot2, reshape2, and viridis. Mutual information and Bayesian prevalence analyses were implemented in MATLAB.

Data and materials availability

Example stimuli, datasets, analysis scripts, and all supplementary materials are available at the Open Science Framework repository: https://osf.io/gtfcn/

Movie S1 contains eight example videos (4 Western European: 2 female, 2 male; 4 East Asian: 2 female, 2 male) illustrating randomly generated facial movements paired with "yes"/"no" utterances in English and Mandarin.

References

1. H. Yu, O. G. B. Garrod, P. G. Schyns, Perception-driven facial expression synthesis. *Computers & Graphics* **36**, 152-162 (2012).

2. J. Zhan, O. G. Garrod, N. van Rijsbergen, P. G. Schyns, Modelling face memory reveals task-generalizable representations. *Nature human behaviour* **3**, 817-826 (2019).
3. A. Röbel, X. Rodet (2005) Efficient spectral envelope estimation and its application to pitch shifting and envelope preservation. in *International conference on digital audio effects*, pp 30-35.
4. A. Röbel, F. Villavicencio, X. Rodet, On cepstral and all-pole based spectral envelope modeling with unknown model order. *Pattern Recognition Letters* **28**, 1343-1350 (2007).
5. C. J. Steinmetz, J. Reiss (2021) pyloudnorm: A simple yet flexible loudness meter in python. in *Audio Engineering Society Convention 150* (Audio Engineering Society).
6. D. Cudeiro, T. Bolkart, C. Laidlaw, A. Ranjan, M. J. Black (2019) Capture, learning, and synthesis of 3D speaking styles. in *Proceedings of the IEEE/CVF conference on computer vision and pattern recognition*, pp 10101-10111.
7. E. Hall, *The Hidden Dimension* (Doubleday, Garden City, NY, 1966).
8. L. Ibrahimagić-Šeper, A. Čelebić, N. Petričević, E. Selimović, Anthropometric differences between males and females in face dimensions and dimensions of central maxillary incisors. *Medicinski glasnik* **3**, 58-62 (2006).
9. N. Newcombe, D. B. Arnkoff, Effects of speech style and sex of speaker on person perception. *Journal of Personality and Social Psychology* **37**, 1293 (1979).
10. J. E. Taylor, G. A. Rousselet, C. Scheepers, S. C. Sereno, Rating norms should be calculated from cumulative link mixed effects models. *Behavior Research Methods* **55**, 2175-2196 (2023).
11. R. A. Ince, A. T. Paton, J. W. Kay, P. G. Schyns, Bayesian inference of population prevalence. *Elife* **10**, e62461 (2021).
12. R. A. Ince *et al.*, A statistical framework for neuroimaging data analysis based on mutual information estimated via a gaussian copula. *Human brain mapping* **38**, 1541-1573 (2017).
13. T. M. Cover, J. A. Thomas, *Elements of information theory* (John Wiley & Sons, 2012).
